# Does Skin-Surface Temperature Reflect Recovery After Total Knee Arthroplasty? A Prospective Pilot Study with Infrared Thermographic Analysis

**DOI:** 10.3390/jcm15207782

**Published:** 2026-10-09

**Authors:** Giacomo Roveda, Domenico Alesi, Federico Maria Adravanti, Luca De Marziani, Giuseppe Filardo, Stefano Zaffagnini

**Affiliations:** 1 Dipartimento di Scienze Biomediche e Neuromotorie (DIBINEM), Università di Bologna, 40136 Bologna, Italy; giacomo.roveda@ior.it (G.R.); luca.demarziani9@gmail.com (L.D.M.); 2 Clinica Ortopedica e Traumatologica II, IRCCS Istituto Ortopedico Rizzoli, 40136 Bologna, Italy; domenico.alesi@ior.it; 3 Facoltà di Scienze Biomediche, Università della Svizzera Italiana, 6900 Lugano, Switzerland

**Keywords:** infrared thermography, total knee arthroplasty, knee osteoarthritis, exercise, inflammation

## Abstract

**Background/Objectives**: Persistent skin-temperature changes have been described following total knee arthroplasty (TKA), but their clinical significance remains unclear. The aim of this study was to compare regional knee temperature before surgery and six months after TKA using infrared thermography. Secondary objectives were to evaluate regional thermal patterns and investigate their association with patient-reported clinical outcomes. **Methods**: Twenty patients undergoing primary TKA for knee osteoarthritis underwent standardized thermographic assessment preoperatively and after six months. Thermograms were acquired at rest (T0), after a two-minute standardized flexion-extension exercise (T1), and after five minutes of recovery (T2). Mean temperatures were calculated for the whole knee and four regions of interest (ROIs). Pain and function were assessed using the Numerical Rating Scale (NRS) and Oxford Knee Score (OKS). Associations between thermographic findings and clinical outcomes were explored. **Results**: Whole-knee temperature was significantly higher at six months than before TKA at all three measurement phases (mean increase, 1.1–1.3 °C; all *p* ≤ 0.002). Exercise induced a transient decrease in temperature both before and after surgery, with no significant change in response magnitude between assessments. NRS pain decreased significantly, whereas OKS improvement was small and did not reach statistical significance (2.15 ± 21.83; *p* = 0.665). NRS improvement correlated inversely with T0 change, most strongly in the lateral ROI (r = −0.903), with greater postoperative temperature increases being associated with less pain improvement. Higher postoperative T2 temperatures were associated with lower OKS scores at follow-up and less OKS improvement across the whole knee and all four ROIs (r = −0.577 to −0.755). **Conclusions**: Skin temperature remained significantly higher six months after primary TKA compared with preoperative values, and higher thermographic values were associated with less favorable clinical outcomes. These preliminary findings, based on exploratory analyses not adjusted for multiple comparisons, support further investigations into infrared thermography as a complementary source of information on postoperative recovery after TKA.

## 1. Introduction

Total knee arthroplasty (TKA) is an effective treatment for end-stage knee osteoarthritis (OA), providing substantial pain relief and functional improvement in most patients [1,2]. Although postoperative healing is generally uneventful, alterations in local perfusion, tissue remodeling, and periarticular inflammation may persist for several months after surgery [3,4,5,6]. Meta-analytic data indicate that the operated knee remains approximately 0.9 °C warmer than the contralateral knee at six months, declining to approximately 0.6 °C at twelve months [7]. Six months therefore represents a point at which thermal abnormality is still clearly measurable, while clinical recovery is largely established. Skin temperature has been investigated as a possible marker of local inflammatory activity [8], although it also reflects tissue remodeling, vascular adaptation, perfusion and autonomic regulation. Growing interest has focused on documenting thermal behavior following TKA.

Studies have reported associations between skin temperature and clinical outcomes after TKA, suggesting that skin-temperature assessment may be clinically informative during recovery [4,9]. Infrared thermography is a non-invasive imaging technique that measures skin-surface temperature and provides indirect information on local perfusion, tissue metabolism, and heat transfer [8]. In symptomatic knee OA, thermographic measurements have been associated with patient and disease characteristics, and regional analysis may reveal patterns not captured by whole-knee measurements alone [10,11]. A single resting measurement describes the joint’s thermal state but not its capacity to respond. Adding a standardized exercise challenge probes the dynamic behavior of the joint, reflecting local vascular reactivity and heat dissipation, and this response has been shown to differ between osteoarthritic and healthy knees [12]. Previous studies showed increased skin temperature following uncomplicated TKA; however, most relied on point measurements, comparisons with the contralateral knee, or assessments performed only at rest [3,4,5,6,13,14]. Windisch et al., for example, described regional temperature differences in the first postoperative week using two regions of interest, without a preoperative reference, a dynamic component, or correlation with clinical outcomes [15]. Consequently, little is known about regional thermal behavior following TKA and its relationship with postoperative clinical outcomes.

The aim of this study was to compare regional knee temperature before surgery and six months after TKA using standardized infrared thermography. Secondary objectives were to evaluate regional thermal patterns and investigate their association with patient-reported clinical outcomes.

## 2. Materials and Methods

### 2.1. Ethical Aspects

This prospective pilot study was conducted at the Clinica Ortopedica e Traumatologica II of the IRCCS Istituto Ortopedico Rizzoli, Bologna, Italy, between January 2023 and September 2025. The study was approved by the Bioethics Committee of the University of Bologna (Ref. No. 283863), and was conducted in accordance with the Declaration of Helsinki. Participation was voluntary, and all participants provided written informed consent before study procedures. The manuscript was prepared in accordance with the STROBE recommendations for observational studies [16].

### 2.2. Study Design and Selection Criteria

Twenty consecutive patients scheduled for primary TKA to treat symptomatic primary knee OA who agreed to participate and provided written informed consent before surgery were enrolled, according to the following criteria. Participation required attendance at a dedicated six-month assessment visit in addition to routine follow-up, and a proportion of eligible patients declined for this reason. Inclusion criteria were: age between 50 and 85 years, clinical and radiographic diagnosis of primary knee OA, and indication for primary TKA. Exclusion criteria were: previous major surgery on the index knee, recent knee trauma or intra-articular injection, inflammatory arthropathy, relevant dermatologic or vascular disease affecting the lower limb, uncontrolled endocrine or metabolic disease, and neurological disorders affecting thermoregulation or exercise performance. Patients experiencing postoperative complications likely to substantially alter local temperature or prevent completion of the standardized assessment were excluded. No patient developed such a complication.

### 2.3. Patient Characteristics

Demographic data including age, sex, body mass index, index-knee side, OA severity (according to Kellgren-Lawrence classification [17]), and symptom duration were recorded. The baseline demographic characteristics of the included patients are summarized in Table 1.

Clinical characteristics of the included patients were evaluated preoperatively and at six-month follow-up using the Numerical Rating Scale (NRS) for pain (range, 0–10) and the 12-item Oxford Knee Score (OKS). The OKS was scored from 0 to 48, with higher values indicating fewer knee-related symptoms and better function [18]. NRS improvement was defined as preoperative NRS minus six-month NRS because lower NRS values indicate less pain. OKS improvement was defined as six-month OKS minus preoperative OKS because higher OKS values indicate better function. The subtraction order therefore differed between the two scales so that positive values consistently indicated clinical improvement.

The sample of 20 patients was selected to provide preliminary estimates of thermal changes and variability, and to evaluate the feasibility of the longitudinal thermographic protocol. These estimates were intended to inform the sample-size calculation of a future confirmatory study.

### 2.4. Surgical Procedure and Postoperative Care

All patients underwent conventional primary TKA through a medial parapatellar arthrotomy. One experienced orthopedic surgeon performed each surgical procedure using a mobile-bearing posterior-stabilized implant. Patellar resurfacing was routinely performed. The standardized rehabilitation pathway comprised progressive weight-bearing to full weight-bearing with crutches and active and passive knee flexion-extension exercises beginning on the first postoperative day.

### 2.5. Infrared Thermography Procedure and Analysis

Preoperative and six-month thermographic evaluations were conducted by two physicians in the same dedicated room, protected from direct sunlight, with temperature held at 23.0 °C by centralized thermostatic control and an average relative humidity of 45 ± 15%. Assessments were performed between 2:00 p.m. and 5:00 p.m. to limit circadian variation. Participants were instructed to avoid prolonged sun exposure during the preceding week, physical activity and analgesic or anti-inflammatory medications during the preceding 48 h, alcohol, smoking, caffeine, cosmetics, or showering during the preceding four hours [19]. Thermograms were acquired with a FLIR T1020 infrared camera (FLIR Systems, Stockholm, Sweden), with a detector resolution of 1024 × 768 pixels and thermal sensitivity of 0.02 °C. Emissivity was set to 0.95 and object distance to 1 m, with reflected apparent temperature set to 20.0 °C; identical camera settings were used at both assessments. The camera was positioned perpendicular to the anterior knee at patellar level and at a fixed distance of 1 m, following the standardized acquisition framework used in prior knee thermography studies [10,12].

Before the first image acquisition, participants exposed the lower limbs and acclimatized in a seated position for 10 min, without clothing or other objects in direct contact with the knee. After the resting thermogram (T0), participants completed two minutes of active knee flexion-extension with a 2 kg ankle weight at one repetition per second, reaching full extension during each repetition. A second thermogram was acquired immediately after exercise (T1). Participants then remained seated under the same environmental conditions throughout recovery. The final thermogram was acquired exactly five minutes after exercise ended (T2). The same protocol was applied at both visits (Figure 1) [12,20]. All participants completed the full exercise protocol at the prescribed cadence and range of motion at both assessments.

Mean temperatures were extracted for the whole anterior knee and four regions of interest (ROIs): medial, lateral, patellar, and suprapatellar. A 2-cm circular marker placed over the center of the patella guided positioning of a standardized digital ROI template on the unmarked thermogram. The patellar ROI consisted of a 6-cm-wide square centered on the patella; the suprapatellar ROI was the 3-cm-high area above it; and the medial and lateral ROIs were the corresponding areas extending 3 cm below the patella on either side. The combined regional area represented the whole anterior knee (Figure 2). Whole-knee temperature was extracted from this combined region as a single ResearchIR selection, yielding an area-weighted average of the four ROIs. Mean ROI temperatures were extracted separately using the same software. All ROI placements and temperature extractions were performed by a single physician using anonymized, randomly ordered thermograms identified only by a numeric code, without access to NRS or OKS data. Complete blinding to assessment timepoint could not be guaranteed, since postoperative images may display surgical scarring [10,12].

Absolute temperature referred to the temperature measured at a specified visit and phase without subtraction. Longitudinal temperature change was defined as the within-patient six-month value minus the corresponding preoperative value at T0, T1, and T2; positive values therefore indicated postoperative warming. Within each visit, the exercise response was defined as T1 minus T0 and the recovery response as T2 minus T0. The longitudinal change in each response was calculated as the six-month response minus the corresponding preoperative response.

The primary outcome was the longitudinal change in mean whole-knee temperature from baseline to six months across the three measurement phases. Exploratory correlation analyses examined associations between clinical outcomes and thermal variables describing the postoperative thermal state, expressed either as the absolute temperature at six months or as the change from the preoperative value, at T0, T1 and T2, for the whole knee and each ROI. Preoperative absolute temperatures and the within-session exercise and recovery responses were analyzed as described above but were not entered into the correlation screening. Given the exploratory design of this pilot study, analyses emphasized effect estimates and variability over hypothesis testing.

### 2.6. Statistical Analysis

Within-patient paired differences showed no substantial departure from normality on graphical assessment and the Shapiro–Wilk test; continuous variables were summarized as mean ± standard deviation. Categorical variables were reported as counts. Paired Student *t* tests were used to compare preoperative and six-month whole-knee temperatures at T0, T1, and T2 and to evaluate within-patient changes in NRS and OKS. Within-visit paired comparisons quantified the exercise response (T1 minus T0) and recovery response (T2 minus T0). The longitudinal change in each response, calculated as the six-month response minus the corresponding preoperative response, was also examined using paired comparisons. Results were reported as mean ± standard deviation and mean paired differences with 95% confidence intervals (CIs).

Associations were evaluated using Pearson correlation coefficients for all 20 participants. Spearman rank correlations were additionally calculated as sensitivity analyses for associations with NRS improvement. Influence diagnostics were computed for each reported association, comprising Cook’s distance and leave-one-out recalculation of the correlation coefficient. Because the analyses were exploratory, the phase and thermal variable producing the largest absolute correlation coefficient for each clinical outcome were identified empirically rather than prespecified. In total, 90 correlations were examined, comprising 30 thermal variables (absolute six-month temperatures and longitudinal changes at T0, T1 and T2, for the whole knee and each of the four ROIs) against three clinical outcomes (NRS improvement, six-month OKS, and OKS improvement).

Because this is a pilot analysis, no multivariable adjustment for potential confounders was performed. Given the multiple exploratory comparisons, correlation analyses were interpreted descriptively and were not adjusted for multiplicity. Statistical significance was set at a two-sided *p* < 0.05. SPSS Statistics version 19.0 (IBM Corp., Armonk, NY, USA) was used for all analyses.

## 3. Results

All 20 enrolled patients completed both the preoperative and six-month assessments, with no loss to follow-up. Whole-knee temperature was higher at six months than before TKA at all three phases (Figure 3). At T0, mean temperature increased from 31.86 ± 1.60 °C preoperatively to 33.11 ± 1.00 °C at six months, corresponding to a mean paired increase of 1.25 °C (95% CI, 0.54–1.96; dz = 0.83; *p* = 0.002). At T1, the mean increased from 31.64 ± 1.52 °C to 32.76 ± 1.08 °C, with a paired increase of 1.13 °C (95% CI, 0.53–1.72; dz = 0.89; *p* < 0.001). At T2, the mean increased from 31.66 ± 1.44 °C to 32.84 ± 1.06 °C, with a paired increase of 1.18 °C (95% CI, 0.60–1.75; dz = 0.96; *p* < 0.001).

Regional temperatures were also higher at six months across all phases (Table 2). Mean paired increases were greatest in the patellar ROI (1.60–1.71 °C), followed by the lateral (1.22–1.45 °C), medial (1.00–1.18 °C), and suprapatellar (0.74–0.81 °C) ROIs.

Whole-knee temperature decreased immediately after exercise at both assessments. Before TKA, the mean exercise response was −0.22 °C (95% CI, −0.43 to −0.01; *p* = 0.037). At six months, the corresponding change was −0.35 °C (95% CI, −0.53 to −0.17; *p* < 0.001). The longitudinal change in the exercise response was −0.12 °C (95% CI, −0.38 to 0.13; *p* = 0.309), indicating that the whole-knee exercise response did not change significantly after TKA.

At T2, whole-knee temperature remained below the resting value. The mean recovery response (T2 minus T0) was −0.20 °C preoperatively (95% CI, −0.43 to 0.04; *p* = 0.094) and −0.27 °C at six months (95% CI, −0.47 to −0.07; *p* = 0.010). The longitudinal change in the recovery response was small and not statistically significant (−0.08 °C; 95% CI, −0.37 to 0.22; *p* = 0.592). Regional exercise and recovery responses followed the same direction in all four ROIs at both assessments, with mean decreases of 0.16 to 0.44 °C after exercise and 0.18 to 0.36 °C at recovery. These regional changes were more variable than the whole-knee response and did not reach statistical significance, with the exception of the six-month medial recovery response (−0.36 °C; *p* = 0.047).

### Clinical Outcomes

Clinical outcomes are summarized in Table 3. NRS pain improved significantly at six months, whereas the mean change in OKS did not reach statistical significance. The change in OKS varied considerably between patients, ranging from −34 to +37 points, with 11 of 20 patients improving.

Exploratory correlations were examined for six-month absolute temperatures and longitudinal temperature changes at T0, T1, and T2. Table 4 presents the strongest associations identified for NRS and OKS outcomes. NRS improvement was most strongly associated with T0 change. The strongest association was observed in the lateral ROI (r = −0.903; *p* < 0.001), followed by the suprapatellar (r = −0.738; *p* < 0.001) and patellar (r = −0.697; *p* < 0.001) ROIs (Figure 4). Because T0 change was calculated as the six-month value minus the preoperative value, these negative coefficients indicate that greater postoperative warming was associated with less improvement in pain. Associations for the whole knee (r = −0.423; *p* = 0.063) and medial ROI (r = −0.317; *p* = 0.174) were not statistically significant. Spearman sensitivity analyses showed the same direction for the lateral, patellar, and suprapatellar ROIs (ρ = −0.891 to −0.730; all *p* < 0.001). The corresponding rank correlations were ρ = −0.543 (*p* = 0.013) for the whole knee and ρ = −0.333 (*p* = 0.152) for the medial ROI.

For OKS, the strongest associations involved absolute postoperative T2 temperature. Across all regions, the postoperative T2 temperature was inversely correlated with both six-month OKS and OKS improvement, an association that was strongest in the lateral ROI (r = −0.698 and r = −0.755, respectively; both *p* < 0.001) (Figure 5). Influence diagnostics indicated that no single observation drove the results: the maximum Cook’s distance across the reported associations was 0.64, below the conventional threshold of 1, and each of the statistically significant associations remained significant after exclusion of any individual participant, with leave-one-out coefficients ranging from −0.495 to −0.922. The complete set of 90 exploratory correlations, with 95% confidence intervals and *p*-values, is provided in Supplementary Table S1; given the exploratory design and the absence of multiplicity correction, all associations should be interpreted as hypothesis-generating rather than confirmatory.

## 4. Discussion

The main finding of this study is that knee skin temperature remained significantly higher six months after primary TKA compared with preoperative values, and that higher thermographic values were associated with less favorable patient-reported outcomes.

The persistence of increased knee temperature observed in this cohort is consistent with previous studies describing prolonged postoperative thermal elevation after uncomplicated TKA. Haidar et al. reported an operated-to-contralateral temperature difference of approximately 0.9 °C at six months [3]. Systematic reviews report the same magnitude at six months, declining to approximately 0.5–0.6 °C at one year [7,21]. These values are not directly comparable with the present results: they express the difference between the operated and unoperated knee at a single timepoint, whereas the present study reports the change from each patient’s own preoperative state. The contralateral knee is frequently affected by osteoarthritis and therefore does not represent a neutral reference, which is one reason the within-patient longitudinal comparison was adopted here. Direct comparison should also be interpreted cautiously because previous investigations generally relied on contact thermometry or single-point infrared measurements [3,4,5,6]. Furthermore, previous studies did not analyze knee regions separately, measuring either a single site or the average of the whole joint, which limits the ability to detect regional differences in thermal patterns [3,4,5,13,14].

The present study extends previous work by showing that higher postoperative skin temperature in several knee subregions was associated with poorer patient-reported outcomes. A persistent local inflammatory response is one plausible contributor to this association, although it is unlikely to be the only one. The association between T0 change and NRS improvement was significant in the lateral, suprapatellar and patellar ROIs but not in the medial ROI or the whole knee, whereas the associations of postoperative T2 temperature with both OKS outcomes were significant for the whole knee and all four ROIs. While this study shows consistent trends, the clinical significance of persistent temperature elevations in specific knee subregions remains to be further explored.

Support for this caution comes from the temporal pattern reported in previous work: systemic inflammatory markers generally return toward baseline within the first postoperative weeks, whereas increased knee temperature may persist for several months after TKA [7,22,23,24]. This dissociation suggests that persistent temperature elevation reflects the combined contribution of several local factors (tissue remodeling, vascular adaptation, skin perfusion, regional autonomic regulation, and prosthesis-related heat transfer), whose relative importance may vary across knee regions and stages of recovery. Future longitudinal studies should investigate these mechanisms by integrating thermographic assessment with inflammatory biomarkers and clinical outcomes.

Further studies could also explore whether implant thermal properties contribute to postoperative skin temperature. Knees replaced with cobalt-chromium and polyethylene components have been shown to generate more heat with activity than knees with ceramic components [25], potentially influencing local heat transfer and patients’ subjective thermal perception. Although the present study was not designed to compare implant materials, the persistence of elevated knee skin temperature observed six months after TKA supports further investigation into whether alternative biomaterials with thermal properties closer to native bone, such as poly-ether-ether-ketone components already under clinical investigation for other TKA outcomes [26], may exhibit different postoperative thermographic patterns. All patients in this cohort received the same implant design, and further studies comparing different implants would be needed to test this possibility.

Exercise-related measurements provide additional information beyond resting temperature by evaluating the dynamic thermal response of the knee. Previous thermographic studies in OA knees and after anterior cruciate ligament reconstruction demonstrated a transient reduction in surface temperature immediately after standardized exercise, followed by gradual recovery [12,20]. This apparently paradoxical decrease is generally attributed to cutaneous vasoconstriction associated with redistribution of blood flow toward working muscle, although direct evidence for this mechanism remains limited. A similar mean response was observed both before and after TKA in the present study, suggesting that, although surgery increased absolute skin temperature, it did not substantially modify the overall physiological thermal response to exercise.

The absence of a statistically significant mean improvement in OKS at six months should be interpreted in the context of the study design. Improvement was heterogeneous rather than absent. This pilot study was not powered to detect change in patient-reported outcomes, and the dispersion observed is consistent with the recognized variability of functional recovery after knee arthroplasty.

The clinical implications of these findings remain exploratory. The observed increase of 1.1–1.3 °C was statistically significant at every measurement phase, but no threshold defining a clinically meaningful temperature difference after TKA has been established, and the present study does not support the use of thermography as a diagnostic tool. Whether standardized infrared thermography can complement clinical examination and patient-reported outcomes in monitoring postoperative joint recovery remains a hypothesis requiring prospective validation in adequately powered cohorts. Whether persistent postoperative temperature elevation reflects prolonged tissue remodeling, altered vascular regulation, implant-related heat transfer, or a combination of these mechanisms remains to be determined.

This study has several strengths, including its prospective design, standardized thermographic acquisition protocol, within-patient longitudinal comparison, regional analysis, exercise-based assessment, and complete follow-up of all enrolled patients. Several limitations should also be acknowledged. This was a single-center pilot study in which all procedures were performed by one surgeon using a single implant design, which limits generalizability. The relatively small sample size limited the statistical power and precluded multivariable analyses, while multiple exploratory correlations increase the possibility of type I error. Reproducibility of region-of-interest placement was not formally assessed, and no intra- or inter-rater reliability data are available. Placement followed a standardized digital template positioned on an anatomical marker, consistent with the protocol used in previous thermographic studies of the knee [10,12,20]. Follow-up was limited to two assessments, preoperative and six months, without intermediate measurements, so neither the trajectory between surgery and six months nor any subsequent thermal normalization could be characterized. The contralateral knee was not imaged. Each patient’s preoperative assessment served as the reference, but imaging the unoperated knee at each visit would have allowed room conditions and body temperature on the day to be accounted for directly. Analgesic and anti-inflammatory medication was withheld for 48 h before each assessment, but habitual use at six months was not recorded. As such medication may influence both skin temperature and reported pain, this represents an unmeasured factor that could have contributed to the observed associations. Participation required an additional study visit at six months, and a proportion of eligible patients declined on this basis. Consent was obtained before surgery and all enrolled patients attended both assessments, so selection did not depend on postoperative outcome; the enrolled cohort may nonetheless differ from the eligible population in baseline characteristics such as availability and willingness to participate in research. Outcomes were limited to patient-reported measures, without objective functional performance testing. Finally, the absence of concurrent imaging or biochemical inflammatory markers limits mechanistic interpretation of the thermographic findings.

Overall, the findings of this pilot study indicate that increased knee skin temperature persists six months after uncomplicated TKA and that higher postoperative temperatures are associated with less favorable patient-reported outcomes. Confirmation of these observations in larger cohorts will help clarify the clinical significance of postoperative thermographic changes and their potential role in evaluating recovery after TKA.

## 5. Conclusions

Knee skin temperature remained significantly higher six months after primary TKA compared with preoperative values, and higher thermographic values were associated with less favorable patient-reported outcomes. These preliminary findings, based on exploratory analyses not adjusted for multiple comparisons, support further investigation of infrared thermography as a complementary source of information on postoperative recovery after TKA.

## Figures and Tables

**Figure 1:**
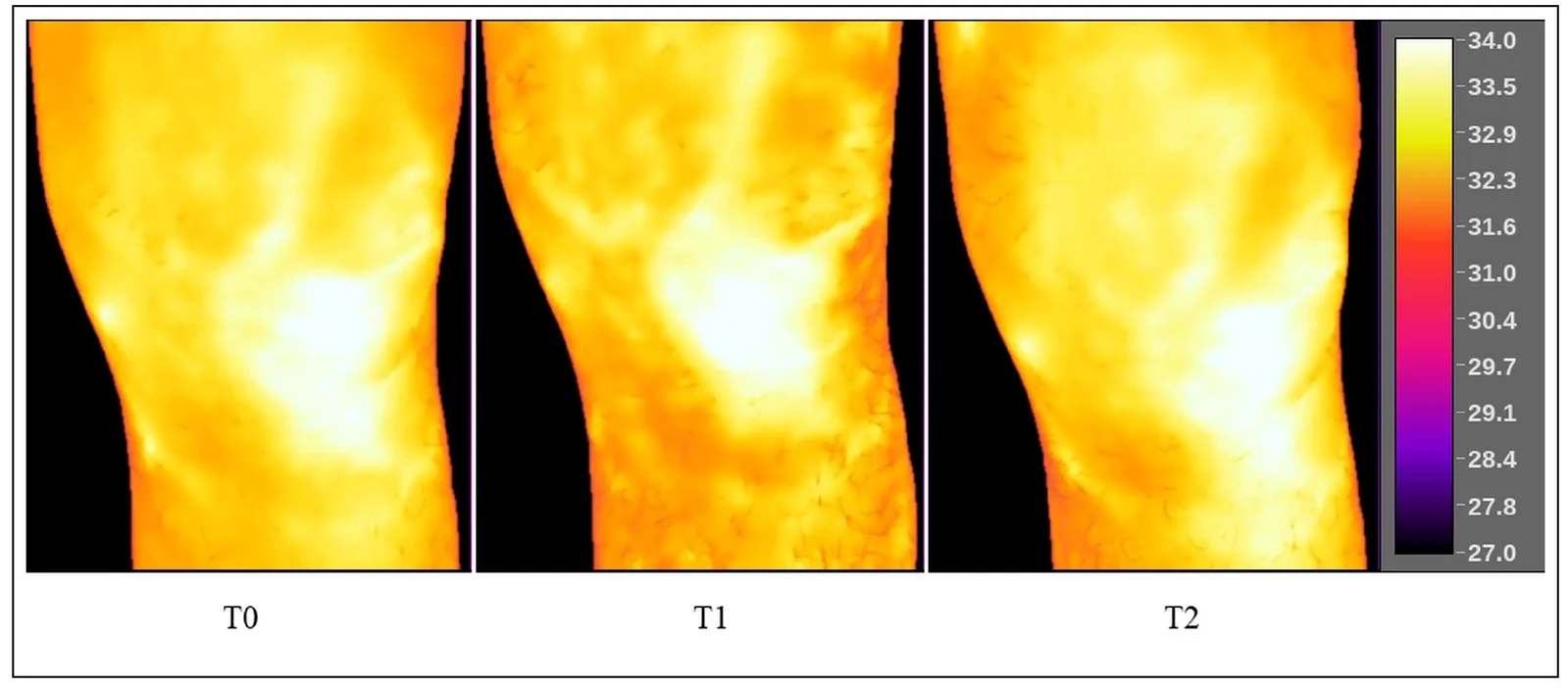
Thermographic acquisition sequence: seated rest before exercise (T0), immediately after the two-minute weighted flexion-extension exercise (T1), and five minutes after the exercise ended (T2).

**Figure 2:**
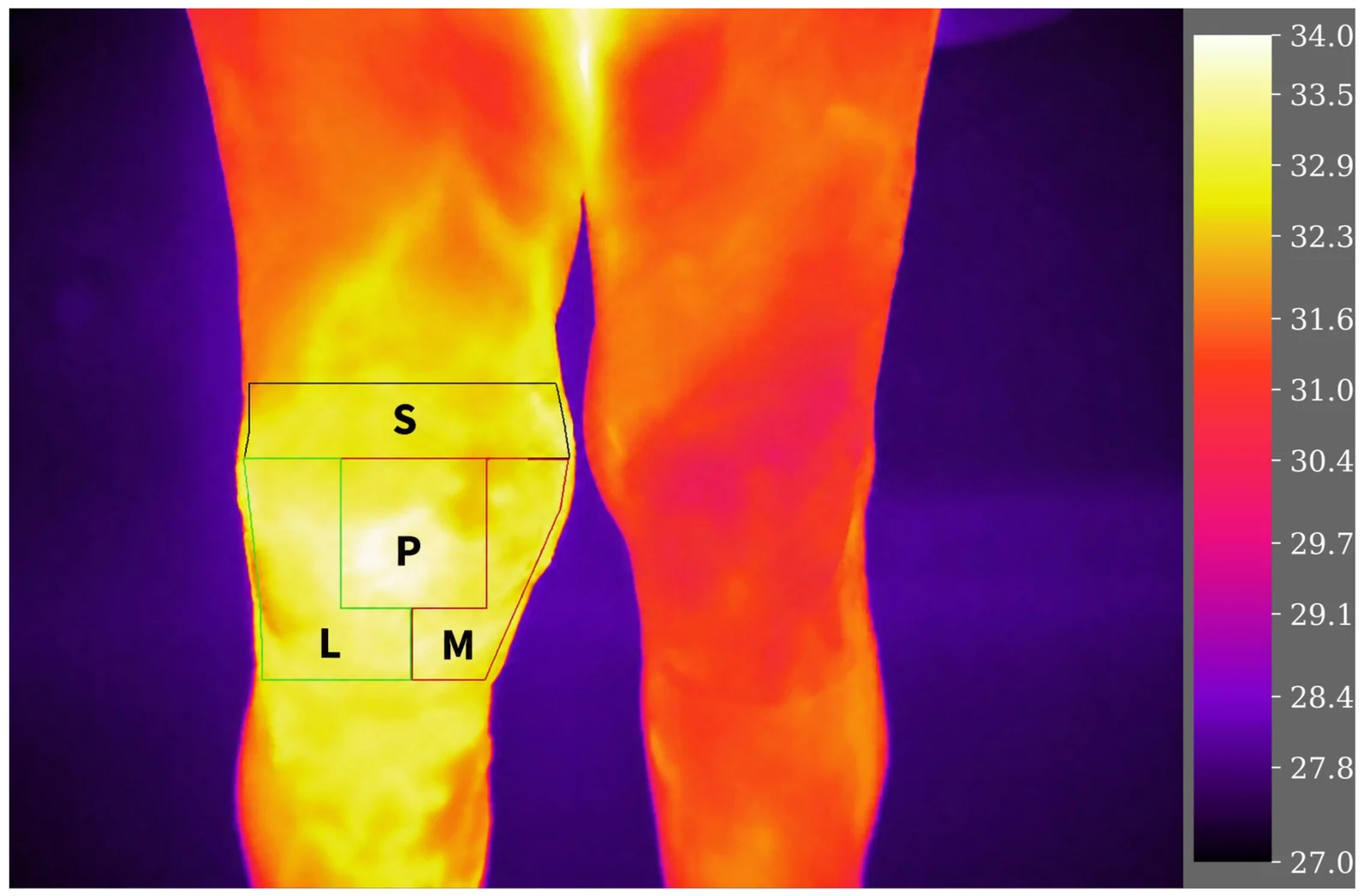
Standardized anterior-knee regions of interest: suprapatellar (S), patellar (P), lateral (L), and medial (M). The four regions were combined into a single selection to obtain the whole-knee temperature as an area-weighted average.

**Figure 3:**
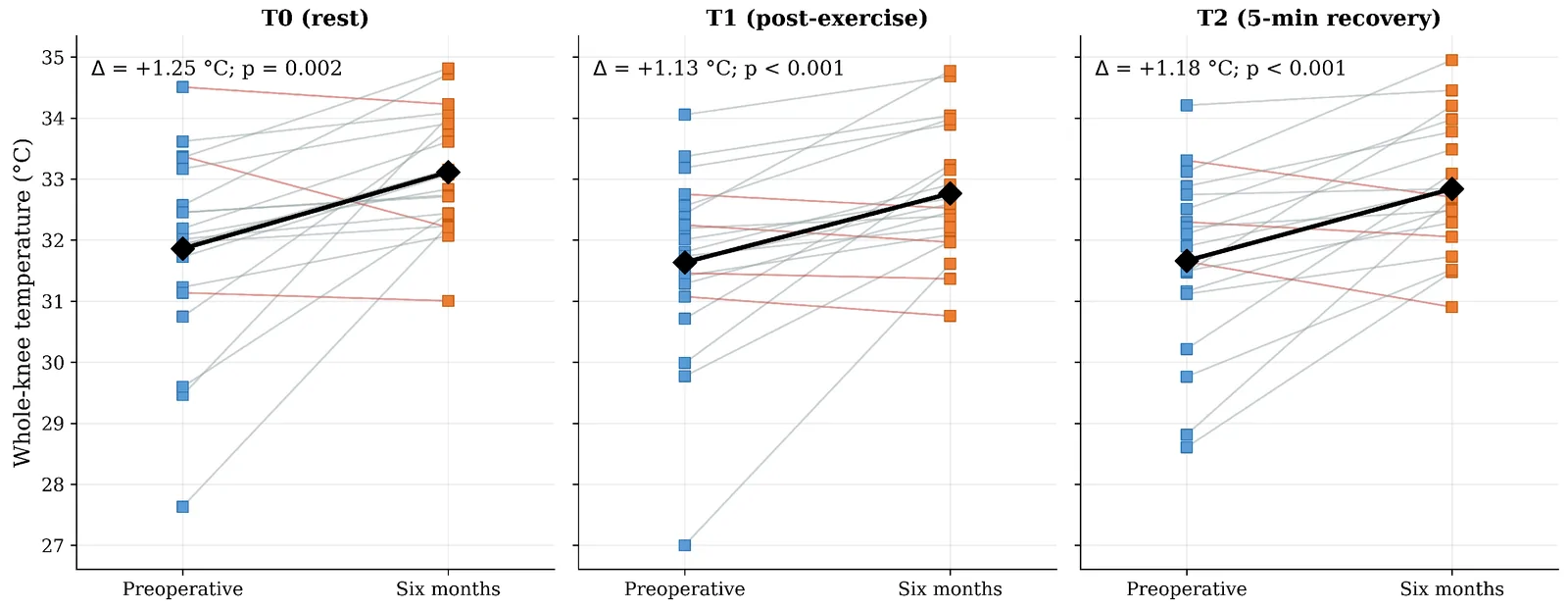
Whole-knee temperature before TKA and at six months at rest (T0), immediately after exercise (T1), and after five minutes of recovery (T2). Lines connect individual patients; red lines indicate patients whose temperature decreased. Black diamonds and the heavy line indicate group means. Δ denotes the mean paired difference.

**Figure 4:**
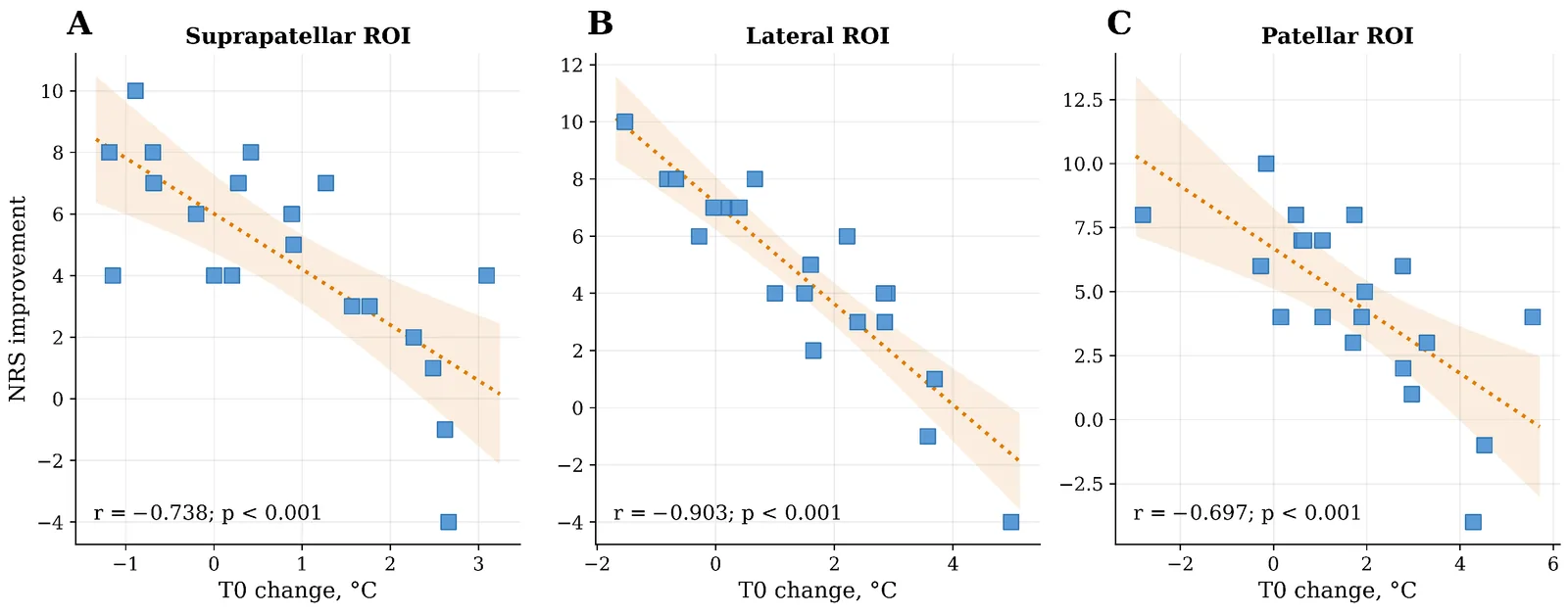
Associations between NRS improvement (preoperative minus six-month NRS) and T0 change (six-month minus preoperative resting temperature). Positive values indicate pain improvement and postoperative warming, respectively. (**A**) Suprapatellar ROI (r = −0.738; *p* < 0.001). (**B**) Lateral ROI (r = −0.903; *p* < 0.001). (**C**) Patellar ROI (r = −0.697; *p* < 0.001). Dotted orange lines indicate linear regression fits with 95% confidence bands.

**Figure 5:**
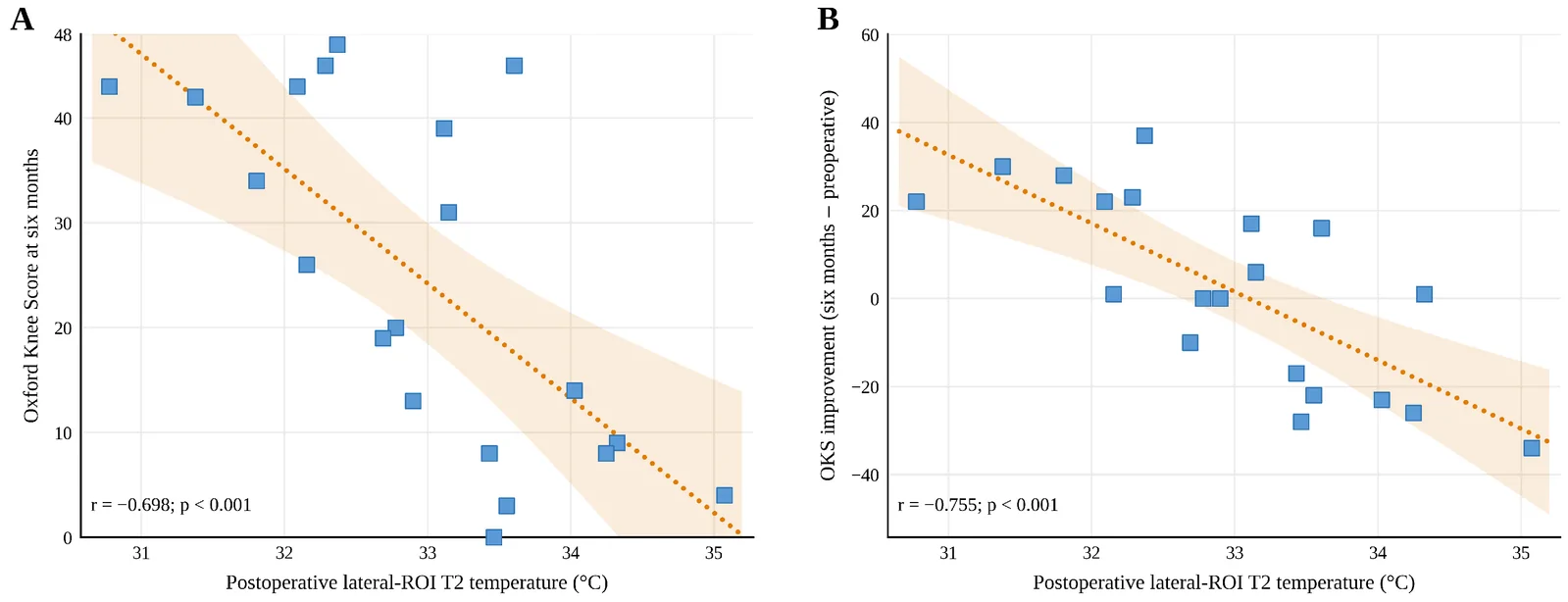
Associations of lateral-ROI postoperative T2 temperature (absolute temperature measured six months postoperatively after recovery) with (**A**) Oxford Knee Score at six months (r = −0.698; *p* < 0.001) and (**B**) OKS improvement (r = −0.755; *p* < 0.001). The Oxford Knee Score ranges from 0 to 48 (higher = better outcome), and OKS improvement was calculated as six-month minus preoperative OKS; therefore, a positive value indicates improvement. Blue markers represent the 20 participants, and dotted orange lines indicate linear regression fits with 95% confidence bands.

**Table 1:** Participant characteristics (N = 20). Values are mean ± SD, range, or number, as indicated.

Characteristic	Value
Age, mean (range), y	68.4 (50–84)
Sex, female/male, n	13/7
Body mass index, mean ± SD, kg/m^2^	24.7 ± 3.6
Operated side, right/left, n	14/6
Kellgren-Lawrence grade III/IV, n	5/15
Symptom duration, mean ± SD, months	23.8 ± 12.4

**Table 2:** Regional temperatures before TKA and at six months. Values are mean ± SD. Paired difference was calculated as six months minus preoperative temperature. CI, confidence interval.

ROI	Phase	Preoperative (°C)	Six months (°C)	Difference (°C)	95% CI	*p*
Suprapatellar	T0	31.98 ± 1.49	32.76 ± 1.04	0.78 ± 1.38	0.13 to 1.43	0.020
	T1	31.68 ± 1.45	32.42 ± 1.20	0.74 ± 1.14	0.21 to 1.27	0.009
	T2	31.76 ± 1.41	32.57 ± 1.23	0.81 ± 1.11	0.29 to 1.33	0.004
Patellar	T0	31.76 ± 1.87	33.47 ± 1.07	1.71 ± 1.93	0.81 to 2.61	<0.001
	T1	31.52 ± 1.77	33.12 ± 1.19	1.60 ± 1.61	0.84 to 2.35	<0.001
	T2	31.57 ± 1.67	33.24 ± 1.08	1.67 ± 1.64	0.91 to 2.44	<0.001
Lateral	T0	31.82 ± 1.67	33.27 ± 1.04	1.45 ± 1.73	0.64 to 2.26	0.001
	T1	31.61 ± 1.57	32.83 ± 1.12	1.22 ± 1.54	0.50 to 1.94	0.002
	T2	31.63 ± 1.50	32.96 ± 1.06	1.33 ± 1.40	0.68 to 1.98	<0.001
Medial	T0	31.85 ± 1.78	33.03 ± 1.17	1.18 ± 1.67	0.40 to 1.96	0.005
	T1	31.69 ± 1.72	32.69 ± 1.20	1.00 ± 1.41	0.34 to 1.66	0.005
	T2	31.67 ± 1.57	32.67 ± 1.19	1.00 ± 1.30	0.39 to 1.61	0.003

**Table 3:** Clinical outcomes before TKA and at six months. NRS improvement was calculated as preoperative minus six-month NRS; OKS improvement was calculated as six-month minus preoperative OKS. Positive values indicate improvement for both outcomes. Values are mean ± SD. dz, Cohen’s d for paired samples.

Outcome	Preoperative	6 Months	Improvement	95% CI	*p*	dz
NRS	7.30±1.59	2.70±2.60	4.60±3.38	3.0 to 6.2	<0.001	1.36
OKS	22.50±9.10	24.65±16.61	2.15±21.83	−8.1 to 12.4	0.665	0.10

**Table 4:** Pearson correlations between regional thermal variables and clinical outcomes, showing for each outcome the thermal variable with the largest absolute coefficient overall, reported for the whole knee and each ROI (N = 20). NRS improvement was paired with T0 change (six-month minus preoperative resting temperature); positive T0-change values indicate postoperative increase in temperature. Six-month OKS and OKS improvement were paired with postoperative T2 temperature (absolute temperature measured six months postoperatively after recovery). NRS improvement was calculated as preoperative minus six-month NRS, whereas OKS improvement was calculated as six-month minus preoperative OKS because lower NRS but higher OKS values indicate improvement.

ROI	NRS Improvement vs. T0 Change, r (*p*)	OKS at 6 Months vs. Postoperative T2, r (*p*)	OKS Improvement vs. Postoperative T2, r (*p*)
Whole knee	−0.423 (0.063)	−0.642 (0.002)	−0.685 (<0.001)
Lateral	−0.903 (<0.001)	−0.698 (<0.001)	−0.755 (<0.001)
Medial	−0.317 (0.174)	−0.611 (0.004)	−0.666 (0.001)
Patellar	−0.697 (<0.001)	−0.590 (0.006)	−0.582 (0.007)
Suprapatellar	−0.738 (<0.001)	−0.577 (0.008)	−0.631 (0.003)

## Data Availability

The data presented in this study are available on request from the corresponding author due to privacy restrictions.

## Supplementary Materials

The following supporting information can be downloaded at https://www.mdpi.com/article/10.3390/jcm15207782/s1, Table S1: Complete set of exploratory correlations between thermal variables and clinical outcomes.

## Abbreviations

The following abbreviations are used in this manuscript:

TKA	Total knee arthroplasty
OA	Osteoarthritis
NRS	Numerical Rating Scale
OKS	Oxford Knee Score
ROI	Region of interest
T0	Resting thermogram
T1	Post-exercise thermogram
T2	Recovery thermogram (5 min after exercise)
SD	Standard deviation
CI	Confidence interval
