# Supplementary material for: Does Skin-Surface Temperature Reflect Recovery After Total Knee Arthroplasty? A Prospective Pilot Study with Infrared Thermographic Analysis
Source: J Clin Med. 2026 Oct 9;15(20):7782. doi: 10.3390/jcm15207782 (PMC13705339; doi:10.3390/jcm15207782)
Supplement: Supplementary file 1 [file jcm-15-07782-s001.zip › jcm-4537380-supplementary.pdf]

Supplementary Table S1

Complete set of exploratory correlations between thermal variables and clinical outcomes (N = 20). All 90 correlations screened are reported. Pearson correlation coefficients with 95% confidence intervals (Fisher z transformation) and uncorrected p-values. Six thermal variables were examined for the whole knee and each of four regions of interest: absolute temperature at the six-month assessment at T0, T1 and T2, and longitudinal temperature change (six-month minus preoperative) at T0, T1 and T2. These were correlated with three clinical outcomes. No correction for multiple comparisons was applied; under Bonferroni correction across all 90 comparisons ($\alpha = 0.00056$) three associations remain significant, indicated in bold.

ROI	Thermal variable	Clinical outcome	r	95% CI	p
Whole knee	Six-month absolute, T0	NRS improvement	−0.291	−0.650 to 0.174	0.213
	Six-month absolute, T0	OKS at six months	−0.532	−0.789 to −0.118	0.016
	Six-month absolute, T0	OKS improvement	−0.576	−0.812 to −0.180	0.008
	Longitudinal change, T0	NRS improvement	−0.423	−0.729 to 0.024	0.063
	Longitudinal change, T0	OKS at six months	0.154	−0.309 to 0.559	0.516
	Longitudinal change, T0	OKS improvement	0.178	−0.287 to 0.575	0.454
	Six-month absolute, T1	NRS improvement	−0.145	−0.552 to 0.318	0.542
	Six-month absolute, T1	OKS at six months	−0.481	−0.761 to −0.049	0.032
	Six-month absolute, T1	OKS improvement	−0.526	−0.786 to −0.109	0.017
	Longitudinal change, T1	NRS improvement	−0.390	−0.710 to 0.064	0.089
	Longitudinal change, T1	OKS at six months	0.043	−0.408 to 0.476	0.859
	Longitudinal change, T1	OKS improvement	0.071	−0.384 to 0.498	0.766
	Six-month absolute, T2	NRS improvement	−0.120	−0.535 to 0.340	0.613
	Six-month absolute, T2	OKS at six months	−0.642	−0.845 to −0.279	0.002
	Six-month absolute, T2	OKS improvement	−0.685	−0.865 to −0.348	<0.001
	Longitudinal change, T2	NRS improvement	−0.485	−0.764 to −0.055	0.030
	Longitudinal change, T2	OKS at six months	−0.041	−0.475 to 0.409	0.865
	Longitudinal change, T2	OKS improvement	−0.034	−0.469 to 0.415	0.887
Suprapatellar	Six-month absolute, T0	NRS improvement	−0.353	−0.688 to 0.107	0.127
	Six-month absolute, T0	OKS at six months	−0.510	−0.777 to −0.088	0.021
	Six-month absolute, T0	OKS improvement	−0.454	−0.747 to −0.015	0.044
	**Longitudinal change, T0**	**NRS improvement**	**−0.738**	**−0.890 to −0.439**	**<0.001**
	Longitudinal change, T0	OKS at six months	−0.266	−0.634 to 0.200	0.257
	Longitudinal change, T0	OKS improvement	−0.128	−0.540 to 0.333	0.590
	Six-month absolute, T1	NRS improvement	0.030	−0.418 to 0.466	0.902
	Six-month absolute, T1	OKS at six months	−0.434	−0.735 to 0.011	0.056
	Six-month absolute, T1	OKS improvement	−0.415	−0.724 to 0.034	0.069
	Longitudinal change, T1	NRS improvement	−0.370	−0.698 to 0.087	0.108
	Longitudinal change, T1	OKS at six months	−0.355	−0.689 to 0.104	0.124
	Longitudinal change, T1	OKS improvement	−0.235	−0.614 to 0.231	0.318
	Six-month absolute, T2	NRS improvement	0.077	−0.379 to 0.502	0.748
	Six-month absolute, T2	OKS at six months	−0.577	−0.812 to −0.181	0.008
	Six-month absolute, T2	OKS improvement	−0.631	−0.839 to −0.261	0.003
	Longitudinal change, T2	NRS improvement	−0.156	−0.560 to 0.307	0.510
	Longitudinal change, T2	OKS at six months	−0.272	−0.638 to 0.194	0.245
	Longitudinal change, T2	OKS improvement	−0.266	−0.634 to 0.200	0.256
Patellar	Six-month absolute, T0	NRS improvement	−0.426	−0.731 to 0.021	0.061
	Six-month absolute, T0	OKS at six months	−0.598	−0.823 to −0.212	0.005
	Six-month absolute, T0	OKS improvement	−0.566	−0.807 to −0.165	0.009
	Longitudinal change, T0	NRS improvement	−0.697	−0.871 to −0.368	<0.001
	Longitudinal change, T0	OKS at six months	−0.284	−0.646 to 0.181	0.225
	Longitudinal change, T0	OKS improvement	−0.194	−0.586 to 0.272	0.413
	Six-month absolute, T1	NRS improvement	0.067	−0.387 to 0.495	0.778
	Six-month absolute, T1	OKS at six months	−0.371	−0.699 to 0.085	0.107
	Six-month absolute, T1	OKS improvement	−0.403	−0.717 to 0.048	0.078
	Longitudinal change, T1	NRS improvement	−0.433	−0.735 to 0.011	0.056
	Longitudinal change, T1	OKS at six months	−0.077	−0.502 to 0.379	0.748
	Longitudinal change, T1	OKS improvement	−0.058	−0.488 to 0.394	0.807
	Six-month absolute, T2	NRS improvement	−0.191	−0.584 to 0.275	0.420
	Six-month absolute, T2	OKS at six months	−0.590	−0.819 to −0.199	0.006
	Six-month absolute, T2	OKS improvement	−0.582	−0.815 to −0.188	0.007
	Longitudinal change, T2	NRS improvement	−0.586	−0.817 to −0.194	0.007
	Longitudinal change, T2	OKS at six months	−0.103	−0.522 to 0.355	0.664
	Longitudinal change, T2	OKS improvement	−0.022	−0.460 to 0.424	0.926
Lateral	Six-month absolute, T0	NRS improvement	−0.448	−0.743 to −0.006	0.048
	Six-month absolute, T0	OKS at six months	−0.505	−0.774 to −0.080	0.023

ROI	Thermal variable	Clinical outcome	r	95% CI	p
	Six-month absolute, T0	OKS improvement	−0.563	−0.805 to −0.161	0.010
	**Longitudinal change, T0**	**NRS improvement**	**−0.903**	**−0.961 to −0.767**	**<0.001**
	Longitudinal change, T0	OKS at six months	−0.384	−0.706 to 0.071	0.095
	Longitudinal change, T0	OKS improvement	−0.365	−0.695 to 0.092	0.113
	Six-month absolute, T1	NRS improvement	−0.175	−0.573 to 0.290	0.459
	Six-month absolute, T1	OKS at six months	−0.469	−0.755 to −0.034	0.037
	Six-month absolute, T1	OKS improvement	−0.476	−0.759 to −0.042	0.034
	Longitudinal change, T1	NRS improvement	−0.597	−0.822 to −0.211	0.005
	Longitudinal change, T1	OKS at six months	−0.223	−0.606 to 0.243	0.344
	Longitudinal change, T1	OKS improvement	−0.161	−0.563 to 0.303	0.498
	Six-month absolute, T2	NRS improvement	−0.252	−0.625 to 0.215	0.284
	Six-month absolute, T2	OKS at six months	−0.698	−0.871 to −0.370	<0.001
	**Six-month absolute, T2**	**OKS improvement**	**−0.755**	**−0.898 to −0.469**	**<0.001**
	Longitudinal change, T2	NRS improvement	−0.605	−0.826 to −0.221	0.005
	Longitudinal change, T2	OKS at six months	−0.425	−0.730 to 0.022	0.062
	Longitudinal change, T2	OKS improvement	−0.413	−0.723 to 0.037	0.071
Medial	Six-month absolute, T0	NRS improvement	−0.258	−0.629 to 0.208	0.271
	Six-month absolute, T0	OKS at six months	−0.441	−0.739 to 0.002	0.052
	Six-month absolute, T0	OKS improvement	−0.485	−0.764 to −0.054	0.030
	Longitudinal change, T0	NRS improvement	−0.317	−0.666 to 0.147	0.174
	Longitudinal change, T0	OKS at six months	0.338	−0.123 to 0.679	0.145
	Longitudinal change, T0	OKS improvement	0.346	−0.114 to 0.684	0.135
	Six-month absolute, T1	NRS improvement	−0.022	−0.460 to 0.425	0.927
	Six-month absolute, T1	OKS at six months	−0.387	−0.708 to 0.067	0.092
	Six-month absolute, T1	OKS improvement	−0.480	−0.761 to −0.048	0.032
	Longitudinal change, T1	NRS improvement	−0.342	−0.681 to 0.119	0.140
	Longitudinal change, T1	OKS at six months	0.295	−0.169 to 0.653	0.206
	Longitudinal change, T1	OKS improvement	0.319	−0.144 to 0.667	0.170
	Six-month absolute, T2	NRS improvement	−0.034	−0.469 to 0.415	0.888
	Six-month absolute, T2	OKS at six months	−0.611	−0.829 to −0.230	0.004
	Six-month absolute, T2	OKS improvement	−0.666	−0.856 to −0.317	0.001
	Longitudinal change, T2	NRS improvement	−0.187	−0.582 to 0.278	0.429
	Longitudinal change, T2	OKS at six months	0.005	−0.438 to 0.447	0.982
	Longitudinal change, T2	OKS improvement	0.006	−0.438 to 0.447	0.980
